# Generation of a protein scaffold for the analysis of functional immunoglobulin epitopes of Bet v 1-like allergens

**DOI:** 10.1186/2045-7022-3-S3-P102

**Published:** 2013-07-25

**Authors:** C Pekar, H Berkner, L Vogel, M Gubesch, L Meisel, S Randow, L Ries, T Holzhauser, J Lidholm, S Vieths, P Rösch, O Hartl-Spiegelhauer, D Schiller

**Affiliations:** 1 Division of Allergology, Paul-Ehrlich-Institut, Langen, Germany; 2 Thermo Fisher Scientific, Freiburg, Germany; 3 Thermo Fisher Scientific, Uppsala, Sweden; 4 Department of Biopolymers, University of Bayreuth, Bayreuth, Germany

## Background

Millions of patients with allergy to tree pollen are sensitized to the major allergen of birch (*Betula verrucosa*) pollen, Bet v 1. Bet v 1-specific IgE cross-reacts with Bet v 1-homologous proteins from plant foods. Only little information on functional IgE epitopes of Bet v 1 and Bet v 1-like allergens in foods is available. We sought to generate a synthetic protein tool to identify and analyze functional immunoglobulin binding sites on Bet v 1 and Bet v 1-like allergens.

## Methods

A synthetic protein (synNCS) was engineered based on the primary structures of both native Norcoclaurine synthase, a non-allergenic protein of the Bet v 1 superfamily, and a natural hypoallergenic Bet v 1 variant, respectively. Recombinant synNCS was purified from *Escherichia coli*. Site-directed mutagenesis was applied to generate synNCS variants carrying potential IgE epitopes of Bet v 1. synNCS and variants thereof were physicochemically characterized using circular dichroism spectroscopy and dynamic light scattering. To evaluate IgE interaction with synNCS, sera of birch pollen allergic patients were tested for IgE binding to purified synNCS in Western blot, ELISA, and cellular mediator release assays.

## Results

Recombinant synNCS variants were expressed in *E. coli*. The theoretical secondary structure topology is similar to Bet v 1. Very low IgE binding to synNCS could be detected in Western blot analyses. However functional *in vitro* IgE interaction with synNCS variants was dependent of the number of potential Bet v 1 IgE epitope residues inserted into the model protein. The synNCS variants induced IgE-mediated mediator release in humanized rat basophil leukemia cells sensitized with sera of birch pollen allergic subjects.

## Conclusion

synNCS, a non-allergenic Bet v 1-like scaffold model protein, could be rendered capable of biologically functional IgE-antibody interaction by insertion of potential Bet v 1 epitope structures into the otherwise IgE-inert synNCS using sera of birch pollen allergic patients. Thus synNCS might be a useful molecular tool to specifically analyze immunoglobulin epitopes of Bet v 1 and Bet v 1-like allergens. The knowledge of determinants of clinically relevant antibody-allergen interactions enables new strategies in the diagnosis, prognosis, and therapy of both tree pollen and pollen-associated food allergies.

## Disclosure of interest

None declared.

